# Effectiveness of Arthroscopic Debridement, Trapeziectomy, and Joint Replacement for Trapeziometacarpal Joint Osteoarthritis: A Meta-Analysis of Pre and Postoperative Pain Scores

**DOI:** 10.7759/cureus.54409

**Published:** 2024-02-18

**Authors:** Kassem Ghayyad, Nikita Golovachev, Nathan Sarli, David Hirsch, Babak Shojaie, Amir R Kachooei

**Affiliations:** 1 Orthopedic Surgery, Rothman Orthopaedics Florida at AdventHealth, Orlando, USA; 2 Plastic and Reconstructive Surgery, Klinikum Bremen-Mitte, Gottingen University of Medical Science, Bremen, DEU; 3 Orthopedics, University of Central Florida, Orlando, USA

**Keywords:** trapeziometacarpal joint osteoarthritis, surgical interventions, pain, systematic review, meta-analysis, arthroscopic debridement, trapeziectomy, joint replacement

## Abstract

Trapeziometacarpal joint osteoarthritis (TMJO) affects up to 33% of postmenopausal women, leading to pain, reduced mobility, and grip strength, with initial treatments focusing on non-surgical options like injections, orthoses, and exercises before considering surgery. A major challenge in managing TMJO involves selecting the optimal surgical strategy that is customized to individual clinical conditions. This study aimed to compare the effectiveness of three common surgical interventions for TMJO in relieving pain, including arthroscopic debridement (AD), trapeziectomy (TRAP), and joint replacement (JR). PubMed, Cochrane, Embase, and MEDLINE databases were queried according to the Preferred Reporting Items for Systematic Reviews and Meta-Analyses (PRISMA) guidelines for studies that presented pain outcomes following intervention for TMJO. Pain scores were reported preoperatively and postoperatively using the visual analog scale (VAS). Inclusion criteria included studies published in Q1 and Q2 journals and those with a follow-up of > six months. The final selection comprised 18 studies with 763 patients treated with AD (n = 102, 13%), TRAP (n = 428, 56%), and JR (n = 233, 31%) between 2010 and 2023, with a mean follow-up period of 38 ± 28 months. The studies included a total of 24 groups, five of which received AD, 13 of which received TRAP, and six of which received JR. The mean preoperative VAS was 6.7 ± 1.7, and the mean postoperative VAS was 1.7 ± 1.3 for all groups (P < 0.001). The meta-analysis demonstrated a mean preoperative pain score of 5.8 (95% CI, 4.1-7.5) for AD, 6.6 (95% CI, 5.7-7.5) for TRAP, and 7.8 (95% CI, 7.0-8.7) for JR. Postoperatively, there was a mean pain score of 2.2 (95% CI, 0.1-4.2) for AD, 1.4 (95% CI, 1.1-1.7) for TRAP, and 0.9 (95% CI, 0.6-1.2) for JR. This study showed that, if appropriately indicated, joint preservation with AD may be as effective as TRAP and JR for reducing pain associated with TMJO in the short term. However, the rate of conversion or revision should be assessed in future studies.

## Introduction and background

Trapeziometacarpal joint osteoarthritis (TMJO) is a common degenerative condition that may significantly impact an individual's quality of life, affecting up to 33% of postmenopausal women [1]. The osteoarthritic changes within the trapeziometacarpal joint lead to pain, limited range of motion, joint instability, and limitations in grip strength, and nonoperative treatment strategies such as intra-articular injections, orthoses, and specific hand exercises are generally pursued first prior to considering surgical interventions [2,3]. Surgical options for the early stages of this condition often aim to preserve the trapezium, utilizing arthroscopic debridement (AD), which involves the removal of damaged or inflamed tissue [4]. In contrast, late stages typically necessitate procedures such as trapeziectomy (TRAP), which involves the complete removal of the trapezium, or joint replacement (JR), where the trapezium is replaced with an implant [4]. The fundamental goal behind these surgeries is to alleviate pain and restore function.

A key challenge in handling TMJO is identifying the most fitting surgical approach tailored to specific clinical situations [5]. This meta-analysis aims to provide an updated perspective on the existing literature, emphasizing comparative studies to discern which among the three techniques - AD, TRAP, or JR - offers the most effective relief in postoperative pain outcomes. We hypothesized that there is no difference in pain level among the three common surgical management techniques of TMJO.

## Review

Materials & methods

This systematic review and meta-analysis adhered to the guidelines outlined in Preferred Reporting Items for Systematic Reviews and Meta-Analyses (PRISMA) [6]. We conducted a comprehensive search for all published clinical studies that presented pain outcomes following TMJO interventions in the following databases: PubMed, MEDLINE, Embase, and the Cochrane Central Register of Controlled Trials (CENTRAL). The literature search was performed in September 2023 using the search strings available in Appendix A.

Eligibility Criteria

Studies that described pre and postoperative pain outcomes using the visual analog scale (VAS) for TMJO, with a minimum follow-up of six months and published in English, were considered potentially relevant. We excluded studies on revision surgeries or nonoperative management, case reports, and review papers. The level of evidence was categorized based on the definition provided by the Oxford Centre for Evidence-Based Medicine [7]. As part of our study, all prospective, randomized, controlled studies (levels I and II), as well as all prospective or retrospective studies with or without control groups (levels III and IV), were included if they recorded the VAS pain score before and after surgery for TMJO.

Study Selection

Our literature review consisted of an electronic search for all relevant articles. We identified 1068 articles that reported clinical outcomes of surgical interventions in patients with TMJO. After removing duplicates, 581 studies remained. Then, the titles and abstracts were screened, and 55 articles met the preliminary inclusion criteria. Lastly, we evaluated the full text of the articles to extract data and found other relevant articles within the reference lists of the papers included in the study. Thirty-one studies remained after a full-text review.

**Figure 1:**
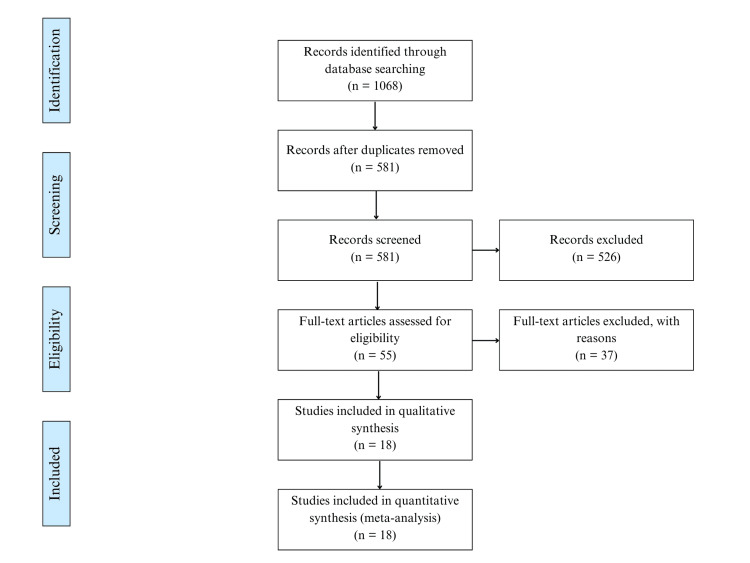
PRISMA flow diagram PRISMA, Preferred Reporting Items for Systematic Reviews and Meta-Analyses.

To ensure the inclusion of high-quality studies, we further filtered the articles based on the journal criteria. Scientific journals are indexed into quartiles (Q1-Q4) based on their impact factor and relevance, with Q1 and Q2 ranking at the top. We enhanced the reliability and credibility of our meta-analysis by including articles published in Q1 and Q2 journals, excluding those from Q3, Q4, and non-indexed sources. After this last stage of filtering, 18 studies published between 2010 and 2023 remained for our analysis (Figure 1). Two independent authors performed the screening criteria, and any conflicts were resolved by a third author.

Outcome Measures

The primary outcome measure was the VAS pain score before and after AD, TRAP, and JR for TMJO. The VAS is a continuous scale that provides a measure of pain intensity [8,9].

Statistical Analysis

Statistical analysis was performed using a random-effects model to calculate the 95% confidence interval (95% CI). The null hypothesis was rejected if the P-value was less than 0.05. To discern the balance between sampling error and true effect, we conducted a heterogeneity assessment using RStudio, focusing on the computation of I^2^. This metric reveals the proportion of dispersion among effect sizes attributable to genuine differences in effects [10]. I^2^ characterizes the percentage of overall variation across studies that results from heterogeneity rather than chance. Negative I^2^ values are adjusted to 0, thereby constraining the I^2^ range between 0% and 100%. An I^2^ value of 0% indicates the absence of observed heterogeneity, while larger values indicate escalating heterogeneity. I^2^ estimates might exhibit unreliability in meta-analyses involving limited trials (e.g., fewer than 10 trials) due to inadequate statistical power [11]. Following a general guideline, we regarded an I^2^ value exceeding 40% as indicative of significant heterogeneity.

Publication Bias

A funnel plot analysis was conducted to determine whether there were any indications of publication bias.

*Quality Control*

The quality of the included studies was evaluated using the Newcastle-Ottawa Quality Assessment Scale [12]. This scale has been used in meta-analysis studies to score the quality of observational studies, including case-control and cohort studies [13,14]. The scale looks at three categories: selection, comparability, and outcome assessment. It is scored from 0 to 9 points, with a higher score indicating better quality.

Results

Study Characteristics

A total of 18 studies were included in the final data extraction. The studies were published between 2010 and 2023, and the mean follow-up period for determining the postoperative VAS pain score in the individual articles was 38 ± 28 months. In terms of the individual surgical treatments, the mean follow-up period was 30 ± 29 months for AD, 33 ± 16 months for TRAP, and 51 ± 30 months for JR (P = 0.287).

There were 763 patients in total, of whom 102 (13%) underwent AD, 428 (56%) underwent TRAP, and 233 (31%) underwent JR. Only one study [15] in this meta-analysis was a randomized control trial (RCT); six studies [16-21] were retrospective cohort studies, six were prospective cohort studies [22-27], four studies were case series [28-31], and one study was case-control [32]. Three studies reported on both JR and TRAP, allowing us to analyze both datasets separately in our meta-analysis [24-26].

After categorizing the studies based on distinct surgical interventions, we identified five groups for AD, 13 for TRAP, and six for JR, resulting in 24 distinct groups for our analysis. In the AD groups, one group performed arthroscopic carpometacarpal (CMC) joint synovectomy and joint debridement [32], while three groups utilized AD with partial TRAP [23,27,30]. In the JR groups, diverse techniques and implants were employed; each group utilized a unique prosthesis [17,21,24-26,28]. In the TRAP groups, the most prevalent technique was ligament reconstruction and tendon interposition (LRTI) using the flexor carpi radialis (FCR) tendon [15,18,22,24-26], followed by other methods such as utilizing suture buttons [15,20,29] and anchoring the abductor pollicis longus (APL) tendon to the articular capsule [22] (Table 1).

**Table 1:** Extracted data from 18 included studies AD, arthroscopic debridement; TRAP, trapeziectomy; JR, joint replacement; EL, Eaton-Littler; D, Dell; VAS: visual analog scale.

Study number	First author	Published year	Journal index	Study design	Treatment modality	Disease staging	Patients (n)	Follow-up (mo)	Mean pre-op VAS	Pre-op SD	Mean post-op VAS	Post-op SD
1	Furia [32]	2010	Q1	Case-control	AD	EL 1 & 2	44	12	7.7	1.4	2.7	1.1
2	Nordback et al. [22]	2012	Q1	Prospective	TRAP	EL mean 2.5 & 2.6	55	12	6.5	3.2	1.07	1.52
3	Taleb et al. [28]	2014	Q2	Case series	JR	EL 2 & 3	7	30	8	1.14	2	4.33
4	Lee et al. [16]	2015	Q1	Retrospective	TRAP	EL 3 & 4	19	36	7.2	1.75	1.7	0.75
5	Pereira et al. [31]	2015	Q2	Case series	AD	D 1, 2, 4	26	20	6.6	1.75	6.03	2.25
6	Chuang et al. [23]	2015	Q2	Prospective	AD	EL 2 & 3	23	24	5.7	0.5	1	0.7
7	Robles-Molina et al. [24]	2017	Q2	Prospective	JR & TRAP	EL 3	65	56	9.24	0.85	1.35	1.84
8	Cebrian-Gomez et al. [25]	2019	Q1	Prospective	JR & TRAP	EL 2 & 3	146	46.6	7.55	1.13	1.06	1.24
9	Oh et al. [26]	2019	Q2	Prospective	JR & TRAP	EL 2 & 3	39	38	6.19	1.76	0.75	1
10	Dreant et al. [17]	2019	Q2	Retrospective	JR	EL 3	25	27.5	8	2.06	1	1.3
11	Lucet et al. [27]	2019	Q2	Prospective	AD	D 2, 3, 4	20	12	2.4	2.9	0.1	0.5
12	Dréant et al. [17]	2021	Q2	Case series	TRAP	EL 4	21	30	3.5	0.75	2	0.75
13	Rodriguez-Buitrago et al. [18]	2021	Q2	Retrospective	TRAP	EL 1, 2, 3, 4	105	8.4	6.95	2.17	1.02	2.01
14	Muramatsu et al. [19]	2022	Q2	Retrospective	TRAP	EL 3	24	20	7.5	0.6	1.8	1.4
15	Zheng et al. [30]	2022	Q2	Case series	AD	EL 2, 3, 4	10	81.6	6.4	1.3	1.1	1.6
16	Yamaura et al. [20]	2022	Q2	Retrospective	TRAP	EL 3 & 4	13	45.4	8.1	1.2	2.9	2.5
17	Morais et al. [15]	2022	Q2	Randomized controlled trial	TRAP	EL 2, 3, 4	76	38.9	4.55	2.71	1.44	1.3
18	Fauqette et al. [21]	2023	Q1	Retrospective	JR	D 2, 3, 4	66	107.5	7.9	1.3	1	1.5

Preoperative VAS Pain Score

**Figure 2:**
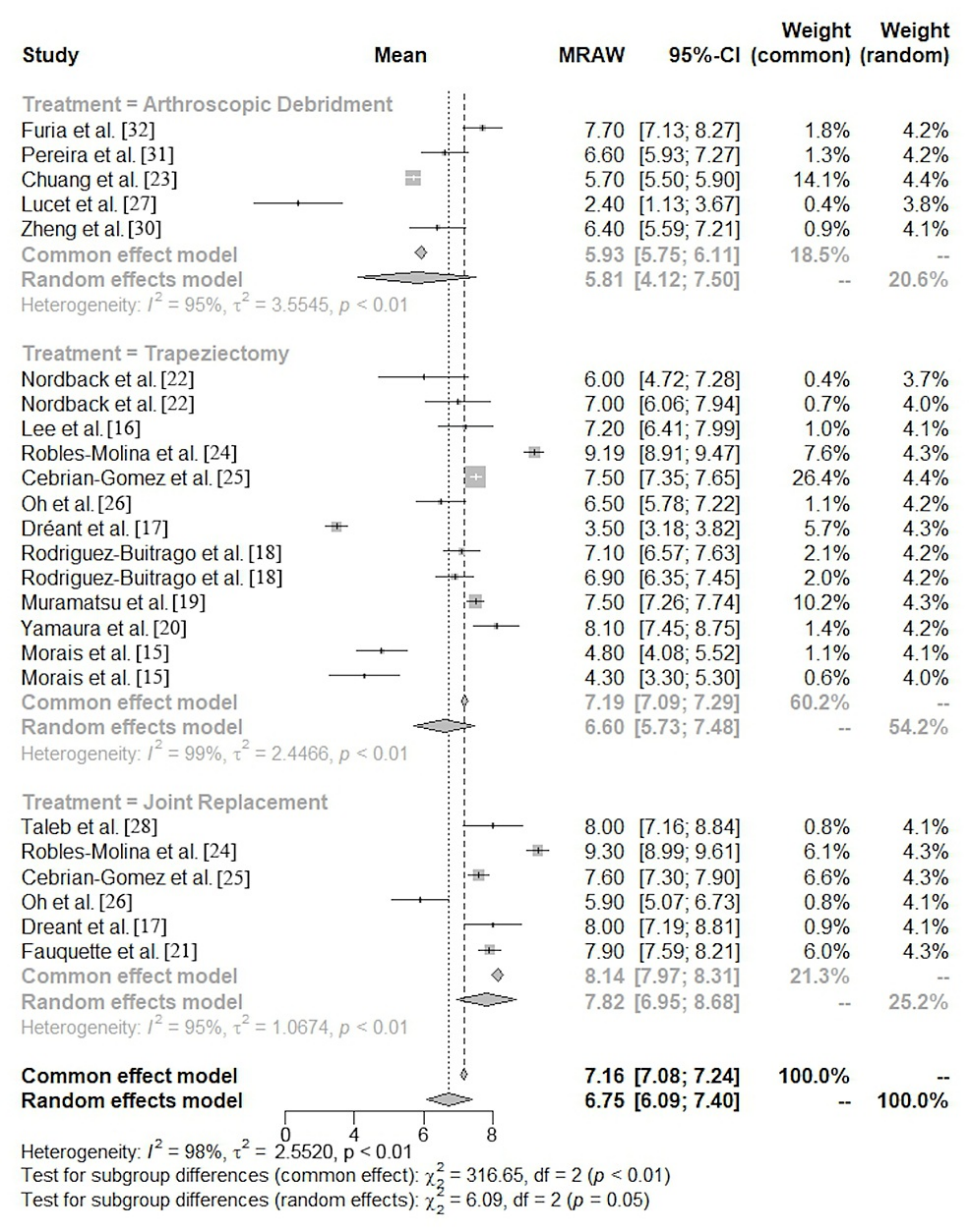
Preoperative visual analog scale forest plot

**Figure 3:**
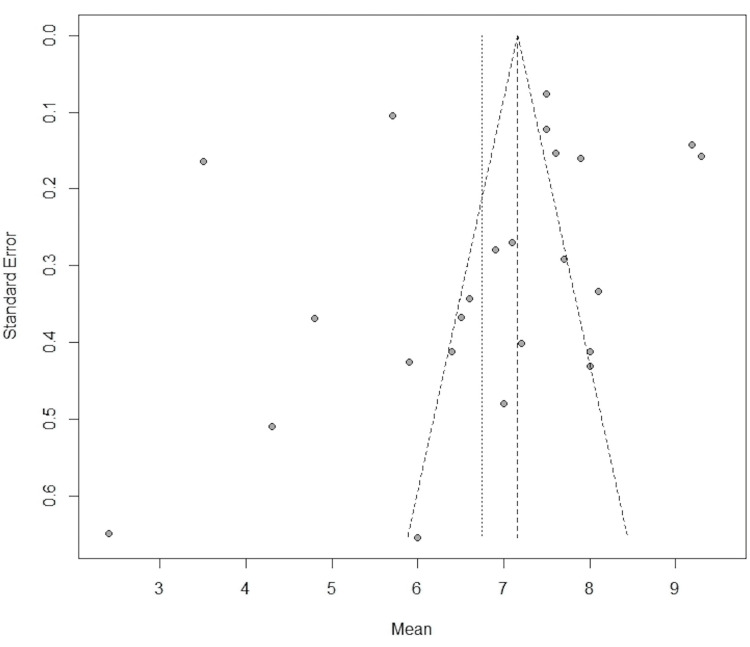
Preoperative visual analog scale funnel plot

The meta-analysis demonstrated a mean preoperative pain score of 5.8 (95% CI, 4.1-7.5) for AD, 6.6 (95% CI, 5.7-7.5) for TRAP, and 7.8 (95% CI, 7.0-8.7) for JR (Figures 2, 3).

Postoperative VAS Pain Score

**Figure 4:**
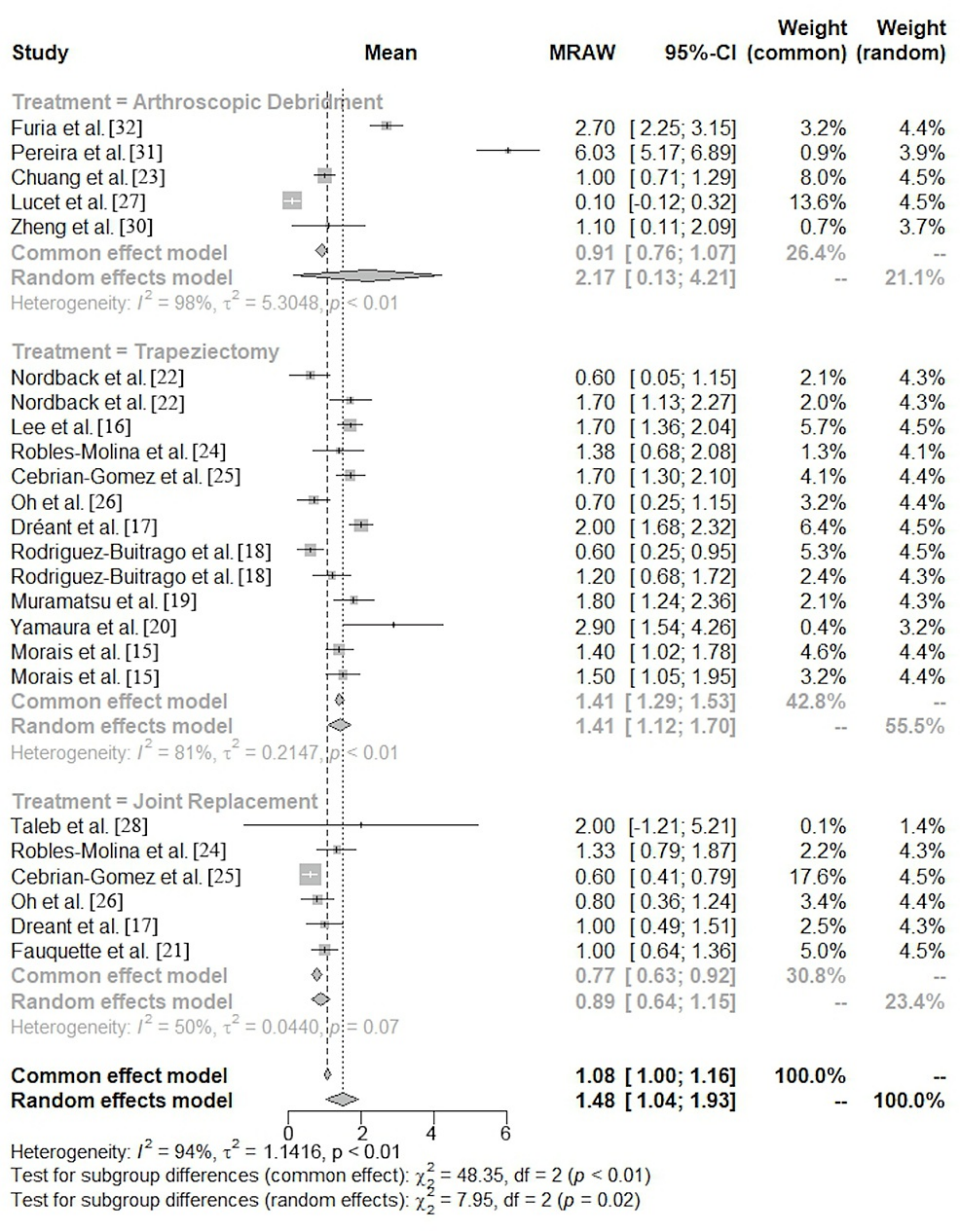
Postoperative visual analog scale forest plot

**Figure 5:**
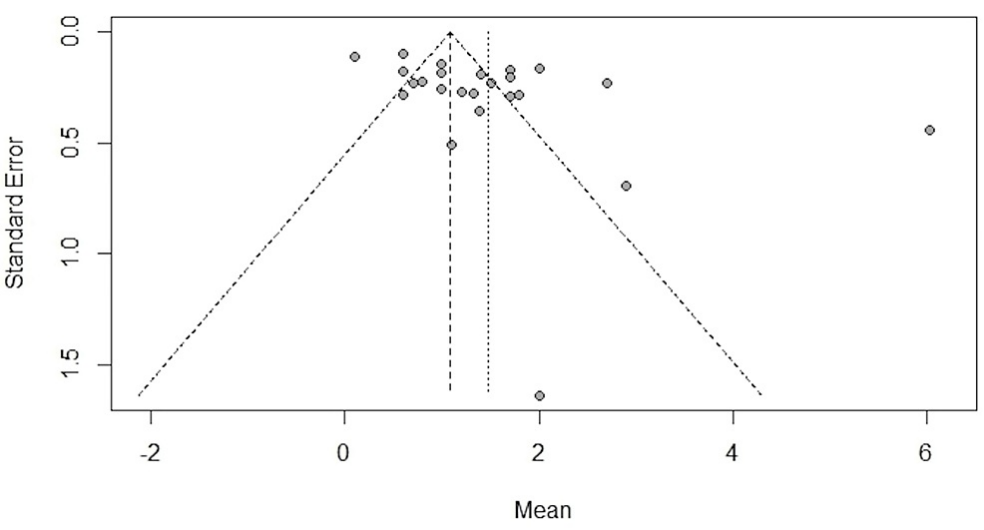
Postoperative visual analog scale funnel plot

The mean VAS pain score postoperatively was 2.2 (95% CI, 0.1-4.2) for AD, 1.4 (95% CI, 1.1-1.7) for TRAP, and 0.9 (95% CI, 0.6-1.2) for JR (Figures 4, 5).

Quality Assessment

The Newcastle-Ottawa Scale score for one case-control study was 9.0 out of 9. The mean score for 12 cohort studies was 7.1 out of 9 (Tables 2, 3).

**Table 2:** Quality assessment of the studies using the Newcastle-Ottawa Scale for case-control studies * A maximum of two stars can be allotted. One for the age and one for the other controlled factors.

Study number	Authors	Published year	Case definition adequate	Representativeness of cases	Selection of controls	Definition of controls	Comparability*	Ascertainment of exposure	Same ascertainment method	Nonresponse rate	Total
1	Furia et al. [32]	2010	*	*	*	*	**	*	*	*	9

**Table 3:** Quality assessment of the studies using the Newcastle-Ottawa Scale for cohort studies * A maximum of two stars can be allotted. One for the age and one for the other controlled factors.

Study number	Authors	Published year	Representativeness of the exposed cohort	Selection of the non-exposed cohort	Ascertainment of exposure	Outcome not present at the start of the study	Comparability*	Assessment of outcome	Follow-up length	Follow-up adequacy	Total
2	Nordback et al. [22]	2012	*	*	*	*	*	*	*	*	8
4	Lee et al. [16]	2015	*		*	*		*	*	*	6
6	Chuang et al. [23]	2015	*		*	*		*	*	*	6
7	Robles-Molina et al. [24]	2017	*	*	*	*	**	*	*	*	9
8	Cebrian-Gomez et al. [25]	2019	*	*	*	*	**	*	*	*	9
9	Oh et al. [26]	2019	*	*	*	*	**	*	*	*	9
10	Dreant et al. [17]	2019	*		*	*		*	*	*	6
11	Lucet et al. [27]	2019	*		*	*		*	*	*	6
13	Rodriguez-Buitrago et al. [18]	2021	*	*	*	*	**	*	*	*	9
14	Muramatsu et al. [19]	2022	*		*	*		*	*	*	6
16	Yamaura et al. [20]	2022	*		*	*		*	*	*	6
18	Fauqette et al. [21]	2023	*		*	*		*	*		5

Publication Bias

We did not observe any visual asymmetry in the funnel plot that would suggest any publication bias (Figures 3, 5). I^2^ demonstrated that most (>90%) of the variation between the studies was due to heterogeneity rather than sampling error. Due to the high heterogeneity of the data, a random-effects model was used to perform the meta-analysis.

Discussion

From our systematic review, we derived data from 18 distinct studies that collectively involved 763 patients and included a varied composition of a randomized controlled trial, cohort studies, case series, and case-control studies. Our study showed no substantial difference between postoperative pain outcomes among the three surgical interventions, indicating that all three interventions can provide acceptable pain relief for TMJO if patients are selected properly.

This study mirrors the scale and scope of previous reviews in the field that examined pain scores after the surgical treatment of TMJO. Raj et al. reviewed 14 studies with 1005 patients to compare TRAP versus JR, finding no significant differences in pain outcomes between the two treatments [33]. Rezzadeh et al. included nine studies with 169 patients to assess the efficacy of TMJO denervation, concluding it effectively provides pain relief [34]. Similarly, Wilkens et al. investigated arthroscopy’s effectiveness in TMJO through 10 studies involving 294 patients, demonstrating significant pain reduction [35]. Our study complements these efforts by providing a balanced and comprehensive examination of surgical interventions for TMJO, contributing to a reliable metric for meta-analytic review. Despite narrowing our search to Q1 and Q2 journals, we believe that our study maintained a sufficient number of studies and sample size, facilitating a thorough evaluation.

A comparison of postoperative pain scores between JR and TRAP has been analyzed by multiple studies [24,25,33,36] and all discerned a clinically insignificant difference in postoperative pain outcomes between the two procedures, except for the most recently published RCT by de Jong et al. [36], who showed that JR was somewhat better in terms of pain reduction than TRAP. However, pain was assessed as part of the Michigan Hand Outcomes Questionnaire and not using the VAS scale, which could explain the significant difference. In the present study, the clinically insignificant difference between pain outcomes between the two procedures was once again noted, persisting for up to 108 months of follow-up [21]. Furthermore, JR and TRAP were found to be applied across various stages of disease progression, with Eaton-Littler staging 2 and 3 being the most common presentations to utilize these techniques [15-22,25,26,28], suggesting that these two techniques can be adapted to different stages of disease severity but favor the middle to later stages.

Rog et al. [37] compared 11 patients who had undergone AD with biological resurfacing to 15 patients who underwent TRAP with LRTI. The TRAP group had statistically significantly better outcomes in VAS scores than the AD group. However, the mean follow-up duration was 15 months for the AD group and 14 months for the TRAP group. The present study demonstrates that at longer follow-up durations, up to 81.6 months in the AD group and 46.6 months in the TRAP group, there are insignificant changes in pain scores postoperatively between the two procedures [25,30]. This observation suggests that while TRAP may offer superior short-term pain relief, the long-term efficacy in terms of pain reduction appears to converge between the two treatments. Additionally, regarding the classification of disease severity through radiographic analysis, TRAP was predominantly selected as a surgical method in the advanced stages of the condition (Table 1). Only one single study chose TRAP for a patient at Eaton-Littler stage 1, representing only 3% of their patient population [18]. Conversely, the use of AD was more commonly applied to the earlier stages of the disease, with the majority of studies including patients within Eaton-Littler and Dell stages 1 and 2 [23,27,31,32].

This study suggests that JR is considered a viable option for more severe disease cases, where joint damage may be too extensive for less invasive treatments such as AD, as JR was heavily favored in patients in more advanced stages of the disease [17,21,24-26,28]. However, three studies in our analysis included patients with advanced arthritis in the AD group (Table 1). In Lucet et al.'s study, the mean preoperative and postoperative pain scores were 2.4 and 0.1. This indicates clinically significant pain reduction, even though initial pain levels were mild across different stages of arthritis [27]. Zheng et al. reported 10 patients with mixed stages of arthritis and substantial pain relief from an average of 6.4 preoperatively to 1.1 postoperatively [30]. However, Pereira et al.’s study contradicted the above arthroscopy results with a high failure rate and conversion to TRAP when arthroscopic intervention was done in the advanced stages of arthritis [31].

In our opinion, proper patient selection and shared decision-making to set expectations are paramount to warrant patient satisfaction. We advocate for nonoperative management as the first-line option for the early stages of the disease, as classified by Eaton-Littler and Dell staging. Should this approach prove ineffective, surgical treatment should be considered, taking into account the costs, recovery times, and potential complications associated with each surgical option. JR has been shown to be associated with greater odds of complications such as loosening, dislocation, and subsequent requirement for revision surgery [33]. AD, on the other hand, was demonstrated to carry the risk of neuropathic pain and paresthesia postoperatively, which may or may not resolve [27]. Additionally, patients undergoing the TRAP technique often experience a decrease in sensation near the surgical scar on the dorsoradial side of the thumb, likely due to injury to the radial nerve branches during surgery [38].

The interpretation of these results should consider the study's limitations. First, we did not analyze the relationship between Eaton-Littler staging and VAS pain outcomes. This was primarily because studies included patients from different osteoarthritis stages. Moreover, there is a poor correlation between the arthritis stage and pain level [39]. Exploring this relationship might have provided insight into how disease staging influences surgical outcomes and subsequent postoperative pain scores. Moreover, the choice of surgery might be influenced by factors such as the orthopedic surgeon’s expertise, patient preferences, age, sex, hand dominance, activity level, or disease stage. Through shared decision-making, the most appropriate surgical approach for each patient can be determined, although our findings may be biased by the retrospective nature of most included studies and the variability in surgical practices. The variability in surgical techniques for TRAP, including the choice between partial and complete TRAP, was also not accounted for in our study and may or may not influence pain outcomes. Additionally, the VAS, though widely used, is inherently subjective. Differences in patient populations, cultural interpretations of pain, and inconsistencies in administering the VAS across studies can also introduce variability in results.

Furthermore, research shows that surgical choices, including the decision between JR and TRAP, may also be influenced by regional practices [40]. Notably, a higher percentage of European surgeons prefer using prostheses in treating TMJO compared to their American counterparts, a difference that might be rooted in the surgeons’ training, local healthcare system structures, and incentives such as health insurance reimbursements [40-42]. Additionally, while the prevalence of arthritis is higher in elderly females, the decision for surgery is primarily based on the impact of pain on daily activities and quality of life, highlighting the complexity of selecting the most suitable surgical approach [43,44].

Future studies should explore factors associated with longevity, functional change, and long-term complications to mitigate variability in surgical options among providers. Because of the poor correlation between pain and the arthritis stage, we believe that the severity of arthritis is not an appropriate indication to offer one intervention over another.

## Conclusions

Despite not examining the association between Eaton-Littler staging and pain scores, we found that while preoperative pain scores varied among the AD, TRAP, and JR procedures, postoperative pain consistently improved. We believe that with appropriate patient selection and discussion of the risks and benefits of each intervention, a satisfactory result can be expected.

## Appendices

Appendix A

Search string used to screen related papers: (thumb basal joint, cmc1, first carpometacarpal) AND (arthritis, osteoarthritis, OA) AND (pain, VAS, visual analog scale).
